# Everything and nothing is conscious: default assumptions in science and ethics

**DOI:** 10.3389/fpsyg.2025.1700354

**Published:** 2026-01-07

**Authors:** Jeff Sebo

**Affiliations:** Department of Environmental Studies, New York University, New York, NY, United States

**Keywords:** consciousness, sentience, null hypothesis, animal welfare, AI welfare

## Abstract

Historically, scientists and philosophers have tended to assume that animals lack consciousness until evidence shows otherwise. Recently, however, some researchers have proposed reversing this assumption. Other options are available as well; for example, in addition to assuming that all animals are conscious, we can assume that all living beings are conscious, that all beings with nervous systems are conscious, that all beings with complex cognition are conscious, or even that all beings are conscious. I examine these options from scientific and ethical perspectives, showing that different default assumptions can be appropriate for different purposes and in different contexts. I also suggest that a default assumption of consciousness may often be best for both science and ethics.

## 1 Introduction^1^

Questions about the distribution of consciousness in the world arise constantly in both science and ethics, requiring us to make assumptions about which beings are conscious and which are not. In science, we must decide whether to attribute subjective experiences to particular entities when interpreting their behavior. In ethics, we must decide whether to attribute subjective experiences to particular entities when making decisions that affect them. These assumptions shape everything from research design and laboratory protocols to farming practices and wildlife management policies. Yet making these assumptions is challenging given the mixed and limited evidence available about nonhuman consciousness, coupled with deep disagreement and uncertainty about what even counts as evidence for beings so different from ourselves.

So, what should we assume about the distribution of consciousness, as we seek to make progress in consciousness science? Traditionally, researchers and policymakers have assumed that nonhuman entities lack consciousness unless sufficient evidence shows otherwise. For researchers, this may be due in part to a preference for false negatives (i.e., Type II errors) over false positives (i.e., Type I errors) (Godfrey-Smith, 1994).^2^ For policymakers, it may be due in part to a preference for maintaining animal use practices that would be problematic if animals were conscious. Either way, the result has been a default skepticism about nonhuman consciousness, which has coincided with a dramatic increase in the scale of human interactions with other animals (Andrews, 2024; Andrews and Huss, 2014; Mikhalevich, 2015; Sober, 2005; de Waal, 1999).

However, some researchers have argued that this default skepticism is unwarranted in science, and have proposed alternatives. One alternative is to revise our default assumptions. For example, Andrews (2024) argues that we should assume that all animals are conscious, and Irina Mikhalevich supports choosing the most evidence-based hypotheses as a default. Another alternative is to reject default assumptions entirely. For instance, Mike Dacey argues that *no* hypothesis should be treated as a default — an approach that he calls “evidentialism.”^3^ And Elliott Sober (2008, 2005) argues that both Type I and Type II errors are bad, and that we can minimize them by avoiding default commitments.^4^ Others propose replacing this methodology with Bayesian (e.g., Kruschke, 2010), likelihoodist (e.g., Anderson et al., 2000), or other approaches.^5^

Some researchers have made similar arguments in ethics as well. For example, Jonathan Birch (2017, 2024) argues that when animals have a realistic chance of being conscious, we should mitigate welfare risks for them. Sebo (2018, 2025) develops this stance through two frameworks: a precautionary principle that supports attributing consciousness, and an expected worth principle that multiplies the probability of consciousness by the potential welfare at stake.^6^ And Knutsson and Munthe (2017) ground this caution in virtue ethics, arguing that moral agents should exercise care when interacting with potentially sentient beings. Together, these approaches suggest that while science may need to revise its skeptical defaults to advance understanding, ethics may need similar revisions to prevent catastrophe.

Assessing these proposals is challenging for at least two reasons. First, the distribution question arises for a vast range of beings. Analyses have traditionally focused on nonhuman animals. However, some researchers are now applying similar analyses to plants, fungi, bacteria, chatbots, robots, emulated minds, brain organoids, and other nonhuman entities. Second, we often combine scientific and ethical discourse and practice together in everyday life, yet different assumptions can make sense for different purposes and in different contexts, both within and across these domains. In science, we can distinguish assumptions that reflect current evidence and those that advance scientific progress in particular contexts. And in ethics, we can distinguish assumptions that ideally balance risks and those that advance ethical progress in particular contexts.

**Figure 1:**
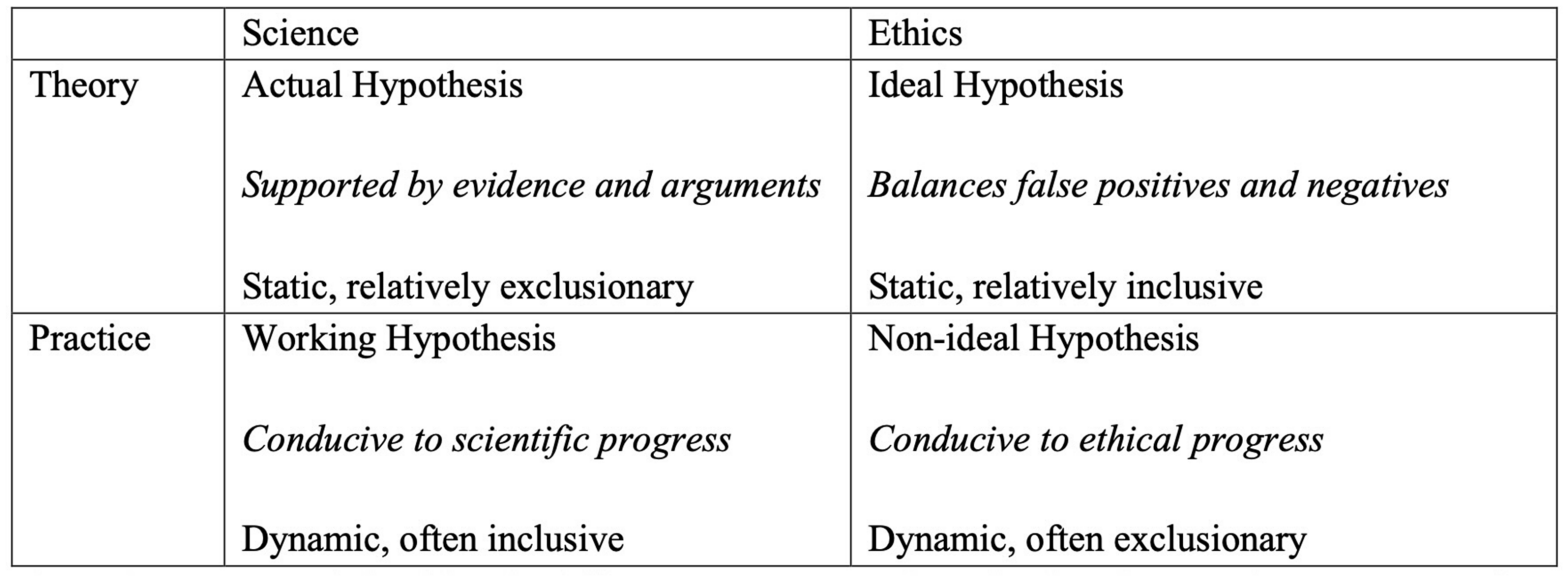
We can and should make different assumptions about the distribution of consciousness for different purposes, including scientific and ethical theory and practice.

This paper argues for distinguishing default assumptions about nonhuman consciousness in scientific theory, scientific practice, ethical theory, and ethical practice, and it maps the terrain across these domains. Section 2 examines different kinds of nonhuman minds for which this question can arise. Section 3 examines default assumptions in science, suggesting that scientific theory may often support more exclusionary assumptions than scientific practice. Section 4 examines default assumptions in ethics, suggesting that ethical theory may often support more inclusive assumptions than ethical practice. Along the way, I argue that clearly distinguishing these analyses makes our task much easier, since it allows us to select complementary assumptions for complementary purposes and improve both science and ethics as a result (Figure 1).

## 2 What should we assume about the distribution of consciousness?

For my purposes here, we can define consciousness as the capacity for subjective experience. If it feels like something to be you, then you are conscious.^7^ This subjective aspect of mind includes both sensory and affective states. Sensory states can include the experience of seeing, hearing, smelling, or touching. Affective states can include positive and negative experiences such as pleasure, pain, happiness, and suffering. While the precise mechanisms underlying consciousness remain the subject of ongoing scientific and philosophical investigation, the basic idea is intuitive: Conscious beings are subjects of experience. In this sense, consciousness is what gives rise to an internal mental life, rather than mere reactivity or information processing.

Determining the distribution of consciousness in the world is important both scientifically and ethically. Scientifically, knowing which beings are conscious helps us better understand what consciousness is and how it arises. This includes gaining insight into the nature and origins of our own consciousness. Ethically, knowing which beings are conscious helps us identify which beings are capable of experiencing pleasure, pain, happiness, suffering, and other morally significant welfare states. This, in turn, informs our moral responsibilities.^8^ If a being can suffer, then they can be harmed, and we have reason to consider their interests when making decisions that affect them. In short, studying consciousness in others helps us not only improve our understanding of them but also our interactions with them.

However, determining the distribution of consciousness in the world is difficult. One challenge is the hard problem of consciousness: the problem of explaining how and why any system can be conscious, including our own brains (Chalmers, 1995). Even if we understand how a system processes information or produces behavior, this is not the same as understanding how or why it feels like something to be that system. Another challenge is the problem of other minds: the problem of knowing what, if anything, it feels like to be another being when the only mind that any of us can directly access is our own (e.g., Carruthers, 2004). We can directly observe behaviors and anatomies, but not thoughts and feelings. These epistemic barriers limit our ability to draw firm conclusions about which beings are conscious.

For much of modern history, many scientists and philosophers regarded the study of nonhuman consciousness as outside the bounds of empirical investigation. Because consciousness is not directly observable, they argued, it could not be studied in the same way as other phenomena (Augustine, 1990, p. 140; Descartes, 1641/2008; Berkeley, 1881, Principle 148). As a result, they often dismissed questions about nonhuman consciousness as nonscientific. Many also assumed that consciousness depends on complex capacities such as language, reason, and self-awareness — capacities that were thought to be uniquely or predominantly human (see, for instance, Carruthers, 2000). On this view, most if not all nonhumans lack the capacity for subjective awareness, and consciousness science lacks the ability to challenge this classification.

Recently, however, emerging research programs have started to make progress on this topic, developing new ways of investigating the distribution question. One approach is the marker method, which allows scientists to make informed probability estimates about nonhuman consciousness based on observable traits (Bayne and Shea, 2020; Birch et al., 2021; Tye, 2016; Varner, 2012).^9^ This method involves distinguishing conscious and nonconscious processing in humans, identifying behavioral and anatomical markers reliably associated with conscious processing in humans, and then searching for similar markers in nonhuman animals. While the presence of such markers might not provide proof of consciousness, it can still serve as evidence, increasing the probability of consciousness under uncertainty (Andrews et al., 2025).

This research has contributed to progress in both science and policy. As demonstrated by the New York Declaration on Animal Consciousness (Andrews et al., 2024), there is now wide agreement that many animals have a realistic chance of being conscious, and that we should consider welfare risks for them. However, there is also a long road ahead. The current evidence is limited and mixed, focused on a relatively small number of species and, in many cases, supporting consciousness in some respects but not others. Moreover, the marker method arguably has inherent limitations, including a bias in favor of confirming evidence and against disconfirming evidence (Andrews, 2024), as well as a bias in favor of humanlike consciousness and against other potential forms of consciousness (Andrews, 2024; Godfrey-Smith, 2020; Kaufmann, 2024; Veit, 2023).

As we continue to make progress in this work, we face an important question: What, if anything, should we assume about the distribution of consciousness as we seek to learn more? We face this question not only with animals but also with a range of other beings, including plants, fungi, bacteria, viruses, chatbots, and robots (Birch, 2024; Sebo, 2025). And in all cases, this choice can influence both how we study consciousness and how we interact with nonhumans under uncertainty. By way of illustrating the complexity of the task, we can here consider five assumptions that we could accept in place of the traditional assumption that nonhumans lack consciousness unless sufficient evidence shows otherwise. These are not the only five options, however, and more work remains to determine which options are most relevant to existing evidence and theory.

**Option 1: All animals are conscious**. Here, we adopt “animal” as the relevant category for the default assumption, and we assume that all animals are conscious unless sufficient evidence shows otherwise. Biologically, animals are often defined as eukaryotic, multicellular organisms that consume organic matter and typically develop the capacity for sensory perception and active movement at some stage of development. This category includes vertebrate groups such as mammals, birds, reptiles, amphibians, and fishes, as well as invertebrate groups like mollusks, crustaceans, and insects. However, it excludes living beings such as plants, fungi, bacteria, and viruses, as well as artificial or non-living entities like robots, chatbots, protons, and electrons (Kushwaha, 2022).

While this option is relatively straightforward, there can be fuzziness at the margins. For instance, sponges are animals by definition, yet they are largely sessile and lack neurons (Adamska, 2016). Conversely, plants and fungi are not animals by definition (and they are also sessile and lack neurons), yet they exhibit surprisingly complex sensory and behavioral capacities (Panda et al., 2025; Braunsdorf et al., 2016). Some organisms also blur categories: Some colonial protists, for example, can coordinate colony-level behavior in ways that resemble simple multicellular animals (Larson, 2023). These examples suggest that the distinction between animals and other living beings might not always be clear or relevant to the distribution question, especially at the margins.

**Option 2: All living beings are conscious**. Here, we adopt “living being” as the relevant category for the default assumption, and we assume that all living beings are conscious unless sufficient evidence shows otherwise. Biologically, living beings are often defined in terms of functions like reproduction, metabolism, and homeostasis, as well as in terms of structures like cells that contain proteins, DNA, RNA, and other biomolecules. On standard interpretations, this category includes animals, plants, fungi, and bacteria, which clearly have the functions and structures characteristic of life. However, it excludes robots (for now), chatbots (for now), tables, chairs, protons, electrons, and a range of other entities that lack these functions and structures (Gómez-Márquez, 2021).

There can be fuzziness at the margins for this option as well. For instance, viruses can reproduce, but only within hosts, and they lack metabolic processes (Gómez-Márquez, 2021). Prions can propagate by inducing other proteins to misfold, but they lack genetic material and metabolic processes (Prusiner, 1984). Researchers in synthetic biology are exploring the creation of “protocells” that can engage in metabolism and self-replication (Deplazes and Huppenbauer, 2009). And researchers in robotics and AI are exploring the creation of silicon-based entities capable of autonomy and self-organization (Aguilar et al., 2014). These examples suggest that the tree of life is like the animal kingdom: The distinction between living beings and other entities may not always be clear or relevant to the distribution question, especially at the margins.

**Option 3: All beings with nervous systems are conscious**. Here, we adopt “a being with a nervous system” as the relevant category for the default assumption, and we assume that all beings with nervous systems are conscious unless sufficient evidence shows otherwise. Nervous systems are often defined as networks of specialized cells that coordinate perceptual inputs and behavioral outputs, typically through the transmission of electrical and chemical signals (Banerjee et al., 2023). On standard interpretations, this category includes all vertebrates and many invertebrates, including relatively simple organisms such as nematode worms. However, it excludes some other invertebrates and many other living beings whose sensory, cognitive, and behavioral systems — if present — are rooted in different kinds of structures.

For this option even more than the previous ones, much depends on how we define our terms. If we set the bar high, requiring structures like neurons and brains, then only vertebrates and many invertebrates would count. If we set the bar lower, recognizing that many chemical or electrical systems can coordinate perception and behavior without neurons and brains, then many plants and fungi might count. We must also ask whether to count only carbon-based nervous systems or to count possible silicon-based analogs too. These boundary cases illustrate that the concept “nervous system” is not always straightforward to apply. However, to the extent that consciousness depends on coordination of perception and behavior, this category might be a more relevant proxy than traditional categories like “animal” or “living being.”

**Option 4: All beings with complex cognition are conscious**. Here, we adopt “being with complex cognition” as the relevant category for the default assumption, and we assume that all beings with complex cognition are conscious unless sufficient evidence shows otherwise. Researchers typically associate complex cognition with a suite of mental capacities, including perception, attention, learning, memory, metacognition, flexible decision-making, and integration of information (Knauff and Wolf, 2010). Depending on how these capacities are defined and measured, this standard plausibly includes many animals and current or near-future AI systems. However, it plausibly excludes many animals and current or near-future AI systems as well, along with plants, fungi, bacteria, viruses, protons, electrons, and other entities.

As with the previous standard, however, much depends on how we define our terms. If we set the bar high — for instance, requiring higher-order thought, language, and reason — then only humans and perhaps future AI systems would count. If we set the bar lower — requiring only the ability to collect, process, and apply information — then many animals, plants, fungi, and other beings might count. Thus, like the “nervous system” option, the “complex cognition” option allows for a wider range of interpretations than the “animal” and “life” options, leaving more room for bias. However, to the extent that consciousness depends on particular kinds of computation and information processing, this category might also be a more relevant proxy than traditional categories like “animal” or “living being.”

**Option 5: All beings are conscious**. This option adopts the broadest possible category — “being” itself — as the relevant category for the default assumption, assuming that all entities that exist are conscious unless sufficient evidence shows otherwise. This idea is most closely associated with panpsychism, the view that consciousness is a fundamental property of matter, much like charge, mass, or spin (Goff, 2017). However, in the same kind of way that an object can be made out of particles that spin without spinning itself, perhaps an object can be made out of particles that feel without feeling itself. Thus, we need to couple this kind of option with a theory of combination — an account of how complex consciousness arises out of simple consciousness — to determine which complex systems have subjective experiences.

As with other options, then, much depends on the details. On a more restrictive interpretation, we might hold that while consciousness is a basic feature of matter, it appears only in select composites, such as living beings with nervous systems or complex cognition. On a more permissive interpretation, we might hold that consciousness exists in all matter, all ordinary objects, and even arbitrary collections of objects, such as the combination of your brain and mine. Like the previous two options, then, the “all beings” option allows for a remarkably wide range of interpretations and applications. Still, for those who see consciousness as a fundamental aspect of reality, this option can seem the most principled and inclusive, offering a straightforward way to avoid the dangers of systematic under-attribution.

We can imagine many other options as well, including options for combining and refining the options already discussed. For instance, we could adopt a restrictive default, such as the view that all and only animals with nervous systems and complex cognition (on restrictive interpretations) are conscious. Or we could adopt a permissive default, such as the view that all living beings, beings with nervous systems, *and* beings with complex cognition (on permissive interpretations) are conscious. These possibilities underscore that the challenge is not merely whether to assume that particular beings are conscious pending further evidence, but also which categories to single out as the relevant ones, and whether our reasons for attributing — or withholding — consciousness by default in some cases extend to others.

## 3 Science

How can we determine which default assumption about the distribution of consciousness is best from a scientific perspective? We can ask this question both in theory and in practice. In theory, the best assumption is the one that best reflects available evidence and arguments (what I will call our *actual hypothesis*). In practice, the best assumption is the one that best drives scientific progress for particular decision-makers in particular contexts (what I will call our *working hypothesis*). If the assumption that seems best in theory is unlikely to spark new questions, studies, or findings, that may be a reason to set it aside for practical purposes. Yet this can also carry risks, since accepting and promoting a working hypothesis that differs from our actual hypothesis could confuse people about the current state of the field.

We can begin by considering the theoretical level of science. Here, one option is an all-or-nothing approach. We start with the assumption best supported by current evidence and theory — for example, “all organisms with nervous systems and complex cognition are conscious.” We then design experiments to test this hypothesis, and we update with sufficient evidence — for instance, by accepting that some organisms *without* nervous systems or complex cognition are conscious. By requiring multiple lines of convergent evidence for updating, this strategy guards against false positives and premature conclusions. As a result, it also makes science more conservative, setting a higher bar for recognizing new entities as conscious and encouraging researchers to focus on a more limited set of familiar possibilities.

Another option is a probabilistic approach. We start by assigning prior probabilities to different hypotheses — for instance, “organisms with nervous systems and complex cognition have a 75 percent chance of being conscious.” We then update these probabilities as new evidence arrives — for instance, assigning an 80 percent chance of consciousness to a particular organism after discovering more sensory or cognitive integration than expected. By modeling assumptions as degrees of belief that can be updated incrementally, this strategy guards against false positives and negatives relatively equitably. As a result, it also makes science more exploratory, setting a lower bar for recognizing new entities as plausibly conscious and encouraging researchers to consider a more expansive set of novel possibilities.

As noted above, while views about the distribution question have varied considerably, many researchers in the twentieth century were skeptical that nonhumans have the capacity for subjective awareness. And to the extent that researchers investigated the possibility of nonhuman consciousness at all, they tended to prioritize nonhuman animals whose behavior, cognition, and evolutionary history are nearest our own. Consequently, early research on nonhuman consciousness tended to focus on other primates, along with other mammals and birds. It also privileged human-centered kinds of evidence, such as linguistic self-report or prefrontal cortical activity. As a result, many researchers defaulted either to a rejection of consciousness in the vast majority of nonhumans or, at least, to a near-zero prior probability.

However, the traditional skeptical assumption about nonhuman consciousness may be too restrictive given the current state of evidence and theory. When we search for evidence with an open mind and non-anthropocentric methods, we tend to find at least some indicators of subjective awareness across a wide range of biological and artificial systems. Additionally, multiple competing theories of consciousness still command substantial support, including theories that attribute consciousness to any system that can process information or represent objects in the environment.^10^ Finally, both the hard problem of consciousness and the problem of other minds limit our capacity to be highly confident one way or the other at this stage. Together these considerations may support a more balanced starting point.^11^

For the all-or-nothing approach, adopting a more balanced starting point would mean accepting more inclusive default assumptions. For example, instead of attributing consciousness only to entities where the evidence is already clear and strong, we might also attribute consciousness to entities that appear relevantly similar to confirmed cases. This approach still requires strong evidence to attribute consciousness, but it also treats apparent relevant similarity as a basis for provisional attribution. As a result, it better balances avoidance of false positives and false negatives, allowing for the possibility of default attribution even when entities have yet to be carefully studied. It also reflects evidence and theory suggesting that consciousness may be much more widely distributed than previously thought.

Similarly, for the probabilistic approach, this would mean assigning more balanced prior probabilities. Instead of treating most entities as almost certainly nonconscious absent strong evidence, researchers can adopt priors that better reflect uncertainty and the diversity of confirmed cases. One possibility is a middle-ground prior, assigning, say, a 25, 50, or 75 percent probability of consciousness to entities that appear relevantly similar to confirmed cases, and then updating with new evidence and theory. Another strategy is an imprecise prior, assigning, say, a 10–90 percent credence range in such cases, and then narrowing or updating the range with new evidence and theory. While these strategies differ, they both imply that we should not default to an extreme view, such as a near-zero or near-100 prior in such cases.^12^

Of course, there are complications. As noted above, the marker method is arguably anthropocentric (Andrews, 2024; Kaufmann, 2024). We increase our confidence that nonhumans are conscious when we find features associated with consciousness in humans, even though consciousness could take other forms too. This method is also arguably asymmetrical. The *presence* of a marker may lead us to *increase* our confidence more than the *absence* of a marker may lead us to *decrease* our confidence, depending on the details. We could have reason to alter our starting points in light of these or other complications. However, it would be a mistake to alter our starting points by rounding all the way up or all the way down; a near-zero or near-100 percent prior still seems too extreme for many of the beings discussed above.

So, when the question is what to assume at the theoretical level of science, where does that leave options like “all animals are conscious,” “all living beings are conscious,” and so on? While a full answer is beyond the scope of this paper, I can say this much at a high level. First, given current levels of disagreement and uncertainty about the distribution question, it helps to take a probabilistic approach rather than an all-or-nothing approach, and to start with a balanced prior probability for most of the categories discussed here. Given that (for better or worse) we treat features associated with consciousness in humans as markers of consciousness in nonhumans, it may also make sense to assign a higher prior probability to beings that appear relevantly similar to humans, by default.^13^

We can now consider this question at the practical level in science. This means asking which assumption will most effectively lead to scientific progress about the nature and distribution of consciousness. For instance, Kristin Andrews argues that flipping the null to “all animals are conscious” would lead to faster progress via better questions, methods, and findings. According to this view, if we focus less on *which* animals are conscious and more on *how* animals are conscious, then we can collect the data needed for a secure theory of consciousness. We can then use this theory to reassess the distribution of consciousness in animals, AI, and other classes of nonhumans. The upshot is that an inclusive default assumption is a practical strategy for overcoming bottlenecks in the science of consciousness (Andrews, 2024).

Of course, a complication is that we should consider more than this kind of generative power when selecting a default assumption regarding the distribution of consciousness. While scientists disagree about how best to select a default, standard practice involves considering a variety of factors, ranging from coherence with evidence and theory to simplicity, testability, tractability, and generativity.^14^ And while these factors might all point in the same direction in some cases (especially for, say, animals with nervous systems and complex cognition), they might point in different directions in other cases (especially for, say, animals without nervous systems or complex cognition, along with plants, fungi, bacteria, viruses, robots, chatbots, and other such entities). If so, careful balancing of different factors may be required.

Consider how theoretical and practical factors can come apart in this domain, particularly for these kinds of edge cases within and beyond the animal kingdom. On the one hand, insofar as the goal is to select a default that best captures existing evidence and theory, it may be best to either deny that sponges, plants, fungi, bacteria, viruses, robots, and chatbots are conscious or, at least, assign a lower probability to these possibilities. On the other hand, insofar as the goal is to select a default that inspires new questions, methods, and findings, it may be best to lean in the other direction in these cases. If so, it may help to clearly distinguish the “actual hypothesis” that we expect to be correct from the “working hypothesis” that we expect to be generative, and to explicitly weigh these theoretical and practical factors together in default assumption selection.

To make matters more complex, the practical factors that bear on default assumption selection can come apart as well, particularly (again) for edge cases within and beyond the animal kingdom. For example, insofar as we value simplicity, testability, and tractability, it may be best to deny that sponges, plants, fungi, bacteria, viruses, robots, and chatbots are conscious when assessing their capabilities. Yet insofar as we value the stimulation of new questions, methods, and findings, it may be best to lean in the other direction — depending, of course, on the specific research question being asked. Here, too, it may help to clearly distinguish the “simplifying assumption” that supports data collection from the “exploratory assumption” that supports innovative thinking, and to explicitly weigh these factors together in default assumption selection.^15^

To make matters more complex still, many other factors bear on the rate of scientific progress as well. To develop a secure theory of consciousness that we can use to assess the distribution question, we may need to consider not only which assumptions and methodologies are simple, testable, tractable, and generative, but also which ones are likely to inspire support for the field. For instance, a research program that adopts a skeptical default stance regarding AI consciousness might appeal to public funders who favor conventional wisdom, but not to private funders who favor innovative thinking. These kinds of cultural factors are rarely made explicit in default assumption selection. However, they clearly influence scientific practice, and they plausibly merit consideration — if not as part of default assumption selection, then at least alongside it.

I have suggested that we can dissolve these tensions to an extent by clearly distinguishing different assumptions that we make for different purposes. However, that might not always be enough to avoid confusion, since nuance can easily be lost in everyday life, and cognitive anchoring can easily make salient ideas appear more plausible, even when nuance is preserved (Furnham and Boo, 2011). Thus, for instance, even if I clearly state that “all living beings are conscious” is a *working* hypothesis, I might still find that my exploration of this idea makes people more credulous about plant, fungi, or bacteria consciousness as an *actual* hypothesis, too. This possibility raises challenging questions about how to resolve tensions between different factors in default assumption selection when all attempts at dissolving them have failed.^16^

Does it follow from any of these complications that, contra Andrews, we should *not* flip the null to “all animals are conscious”? Not at all. Instead, it simply follows that null hypothesis selection — and, more generally, *default assumption* selection in science — is complex. It requires assessing a wide range of candidate assumptions from a wide range of theoretical and practical perspectives, taking care to distinguish different kinds of assumptions where possible and weigh different kinds of factors together where necessary. Either way, I agree with Andrews about the generative power of attributing consciousness to most if not all animals. And when we clearly distinguish generative power from other factors, we may find that a similar analysis extends, to greater or lesser degrees, to many entities outside the animal kingdom too.

So, when the question is what to assume at the practical level in science, where does that leave options like “all animals are conscious,” “all living beings are conscious,” and so on? Much depends on context. For some purposes — such as extending established research agendas or appealing to traditional funders — exclusionary assumptions may often be most effective. For others — such as starting new research agendas or attracting alternative funders — inclusive assumptions may often be most effective. Still, I agree with Andrews that asking *how* nonhumans are conscious, assuming that they are, is an effective way to make progress in many cases. I thus expect that our working hypotheses should be dynamic and often inclusive, since we should vary them across contexts and often use them to explore new ideas.

To take stock: I have suggested that we should adopt different assumptions about the distribution of consciousness for different purposes in science. At the theoretical level, our defaults should reflect what we expect to be true given current evidence and arguments. At the practical level, they should reflect what we take to be useful for advancing science. I have also suggested that our theoretical assumptions about the distribution question should generally be more static and exclusionary, while our practical assumptions should generally be more dynamic and exploratory. Still, if we adopt a probabilistic approach rather than an all-or-nothing approach, then these approaches may differ in degree, not in kind, since a middle-ground prior probability may be appropriate for most of the categories discussed here.

## 4 Ethics

How, then, can we determine which default assumption about the distribution of consciousness is best from an ethical perspective? Here too we can distinguish between theory and practice. In theory, the best assumption is the one that best balances risks of false positives and negatives about consciousness (what I will call our *ideal* hypothesis). In practice, by contrast, the best assumption is the one that best drives moral progress in particular contexts, taking into account how likely people are to act on it (what I will call our *non-ideal* hypothesis). If the assumption that seems best in theory is unlikely to gain traction in practice, that may be a reason to set it aside, at least temporarily. Yet this can also carry risks, since delaying recognition for potentially conscious beings could mean allowing preventable harms to continue.

We can begin by considering the theoretical level of ethics. As before, we can distinguish between all-or-nothing and probabilistic approaches. The all-or-nothing approach, which Sebo (2018, 2025) describes as *the precautionary view*, holds that we should seek to avoid worst case scenarios. In this context, that means comparing the magnitude of harm associated with false positives (how bad would it be if we mistakenly treated nonconscious beings as conscious?) with the magnitude of harm associated with false negatives (how bad would it be if we mistakenly treated conscious beings as nonconscious?). We then adopt the assumption that avoids the greater harm. For example, if mistakenly treating ants as conscious would be more damaging than mistakenly treating them as conscious, then we treat them as conscious.

By contrast, the probabilistic approach, which Sebo (2018, 2025) describes as *the expected weight view*, holds that we should maximize expected value. In this context, that means comparing the probability and magnitude of harm associated with false positives (how likely are over-attributions of consciousness, and how harmful would they be?) with the probability and magnitude of harm associated with false negatives (how likely are under-attributions of consciousness, and how harmful would they be?). We then distribute moral concern to particular beings accordingly. For instance, if ants have a 10% chance of being conscious and can suffer 10% as much as humans if they are, then we should treat the worst apparent ant pain as roughly 1% as bad as the worst apparent human pain when evaluating trade-offs.

While ethical practices vary, the dominant approach in the West has been to treat nonhumans as nonconscious by default. In the first half of the twentieth century scientists were reluctant to engage with the topic in ways that might challenge this stance (e.g., Watson, 1928; Skinner, 1953), and policymakers generally followed suit. By the latter half of the century, however, scientific attitudes began to shift: Researchers first allowed that nonhuman primates are likely conscious (e.g., Gallup, 1970), then other mammals (e.g., Seth et al., 2005), then birds and certain other animals (e.g., Cabanac et al., 2009). Policymakers then *sometimes* followed suit, typically treating attributions of consciousness as a necessary but not sufficient condition for extending welfare protections.

From the standpoint of ethical theory, however, this default skepticism is problematic under both the precautionary view and the expected weight view. According to the precautionary view, since our aim is to avoid worst case scenarios, we should often attribute consciousness by default. For animals in particular, the harm of false positives is that we treat objects like subjects unnecessarily, forming inappropriate bonds with them and diverting resources away from actual subjects who need them. The harm of false negatives, by contrast, is that we treat subjects like objects unnecessarily, potentially causing or allowing them to suffer at vast scales and for trivial reasons. While both mistakes are bad, the mistake of treating subjects like objects is often far worse than the mistake of treating objects like subjects.

Does this mean false negatives are always worse for the precautionary view? Not necessarily. In some contexts, false positives could be *extremely* bad. For instance, over-attributing AI consciousness could be much worse than over-attributing animal consciousness. If we came to believe that future AI systems were conscious, then we might feel pressure to grant them political rights. The result could be human disempowerment, perhaps even extinction^17^ — all for the sake of entities with no inner mental life. Even a low but nonnegligible chance of such an outcome (about which more in a moment) could be enough to make the harm of over-attribution rival the harm of under-attribution (Long et al., 2024). Still, the broader point remains: A policy of default skepticism is *often* problematic for the precautionary view.

Now take the expected weight view. If our goal is to maximize expected value, then we should likewise sometimes default to attributing consciousness, with varying degrees of moral concern to match our varying degrees of confidence about consciousness. Indeed, according to one interpretation of this view, we should attribute at least *some* consciousness (even if only very little) to all entities with a nonzero chance of being conscious. Given the current state of consciousness science, this view could imply that we should attribute at least some consciousness to, if not all entities in the universe, then at least very many of them, including vertebrates, invertebrates, plants, fungi, bacteria, viruses, chatbots, robots, and perhaps even parts or collections of these beings, such as brain regions or insect colonies.

Granted, other interpretations of this view set a higher bar, holding that we should attribute at least some consciousness to all entities with a *nonnegligible* chance of being conscious. What counts as a nonnegligible risk, according to such views? Answers to this question tend to range between a *one in ten quadrillion* risk and a *one in ten thousand risk* (Monton, 2019). However, while these views might diverge with respect to some cases, they converge with respect to attributions of at least *some* consciousness to all entities with at least, say, a *one in a thousand* chance of being conscious — a standard which many entities plausibly meet (Sebo and Long, 2023; Sebo, 2025). In any case, the present point is once again simply that a policy of default skepticism is often, and perhaps *very* often, problematic for the expected weight view as well.

So, when the question is what to assume at the theoretical level of ethics, where does that leave options like “all animals are conscious,” “all living beings are conscious,” and so on? While a full answer is beyond the scope of this paper, I can say this much at a high level. We should attribute at least some consciousness, even if only very little, to many of the beings discussed in this paper according to the expected weight view, given current evidence. And when the potential harm associated with false negatives is clearly worse than the potential harm associated with false positives — a condition that plausibly holds in a wide range of cases, even if it may be more likely to hold for some beings, like nonhuman animals, than for others, like AI systems — then we should do the same according to the precautionary view.

We can now turn to the practical level in ethics. Even if it would be ideal for us to treat sextillions of beings as at least minimally conscious in principle, it may not always be ideal for us to accept, promote, or apply this assumption in practice. After all, significant limitations on our knowledge, power, and motivation shape what we can realistically achieve and sustain. When deciding which assumptions to adopt, then, we must consider not only which ones are best for managing risk all else being equal, but also which ones are most likely to be achieved and sustained by particular agents in particular contexts and build momentum toward a better calibrated moral circle. And while a more inclusive assumption might often be the better principled stance, a more exclusionary assumption might sometimes be the better pragmatic stance.

There are many reasons why theory and practice might come apart in this regard. Consider two. First, we tend to be better at applying simple rules than complex ones in practice. Thus, for instance, even if one thinks that assigning a different chance of consciousness to each species is ideal in theory, one might still think that taking a simpler approach — say, assigning a 90% chance to mammals, an 80% chance to birds, a 70% chance to other vertebrates, a 60% chance to cephalopods and decapods, a 50% chance to other invertebrates, and so on — is best for some purposes in practice. The reason is not that these estimates ideally balance the risk of false positives and negatives, but rather (one might think) that they provide the closest approximation to this ideal that ordinary agents can be expected to reliably apply in everyday life.^18^

Second, we also tend to be better at implementing incremental changes than at implementing transformative ones.^19^ Thus, for instance, even if one thinks that attributing consciousness to all animals, plants, fungi, bacteria, viruses, chatbots, robots, and so on is ideal in theory, one might still conclude that taking a more incremental approach — for example, attributing at least minimal consciousness to all vertebrates, many invertebrates, and some near-future AI systems as a starting point — is ideal in practice. Again, the reason is not that this more modest assumption about the distribution of consciousness ideally balances the risks of false positives and negatives, but rather that it represents the greatest amount of moral circle expansion that agents and institutions can realistically be expected to accept at present.

Does that mean that we should always accept or promote simple, moderate assumptions in practice? Not necessarily. It can often be helpful to accept relatively complex and radical assumptions oneself while promoting relatively simple and moderate ones to others (Sebo, 2023). Additionally, it can often be helpful to promote complex and radical assumptions in some contexts (say, when talking with fellow researchers) while promoting simple or moderate ones in others (say, when talking with companies and governments). And it can often be helpful to adopt a division of labor, where some people promote complex or radical assumptions to shift the center of debate and pave the way for incremental reform, and others promote simple or moderate ones to shift the goal posts and pave the way for transformative change.

Of course, a challenge for ethics — as for science — is that theory and practice can be easy to conflate in many cases. Suppose I argue that we should treat all living beings as at least minimally conscious. Even if my intention is to make this argument at the level of theory, asserting that all living beings merit at least minimal moral consideration, people might interpret me as making it at the level of practice, advocating for immediate and transformative policy change. Conversely, suppose I argue that only vertebrates and some invertebrates should be treated as minimally conscious. Even if my intention is to make this argument at the level of practice, proposing policies that can be adopted today, people might interpret me as making it at the level of theory, denying that other beings merit even minimal moral consideration.

More generally, default assumptions in science and ethics are themselves easy to conflate. Suppose I argue that insects or near-future AI systems merit moral consideration. If my argument reaches a wide enough audience, I can expect some of its nuances to disappear, with some people hearing me as arguing that these beings are either conscious or, at least, *probably* conscious. Conversely, suppose I argue that these beings are either nonconscious or, at least, *probably* nonconscious. Here I can expect the same distortion in reverse, with some people hearing me as denying that these beings merit moral consideration. In all cases, these conflations — involving assumptions made for scientific theory, scientific practice, ethical theory, *and* ethical practice — must be carefully managed.

While I will not be able to say here how best to weigh these factors, I will make at least one comment for now, without fully defending it. In my view, none of the levels discussed here should take lexical priority over the others in cases of conflict. For instance, it may be tempting to say that scientific theory should take lexical priority over ethics and practice. But science, ethics, theory, and practice are too interwoven for such a simple policy, and there are many cases where simple approximations of the truth (say, “all animals have a realistic chance of being conscious”) are best not only for making scientific and ethical progress but also for *bringing societies closer to the truth*. In these and other such cases, it seems like a clear mistake to focus on the evidence alone, making zero concessions to ethics or practice.

So, when the question is what to assume at the practical level in ethics, where does that leave options like “all animals are conscious,” “all living beings are conscious,” and so on? As in science, much depends on context. Suppose I believe that, in principle, we should attribute at least minimal consciousness to all the beings discussed in this paper. What follows for my everyday decisions may be complex. For purposes of making decisions as an individual, perhaps I should accept the closest approximation to the ideal hypothesis that I can be expected to internalize. But for purposes of outreach to others, perhaps I should try to meet different audiences where they are, promoting the closest approximation that they can be expected to accept at present, while still keeping the door open for further expansion over time.

To take stock: I have suggested that we should adopt different assumptions about the distribution of consciousness for different purposes in ethics, as in science. At the theoretical level, our defaults should ideally balance the risk of false positives and the risk of false negatives. At the practical level, our defaults should also reflect what particular agents are able to achieve and sustain at present and what will build momentum toward a better calibrated moral circle in the future. As in science, our assumptions in ethics will tend to be more static in theory and dynamic in practice, given the contextual nature of practical decision-making. Yet unlike in science, our assumptions in ethics may be more inclusive in theory and more exclusionary in practice, reflecting the gap between “ideal” and “non-ideal” approaches to ethics.

## 5 Conclusion

I have argued that we should select different default assumptions about the distribution of consciousness for different purposes and in different contexts, both within and beyond the animal kingdom. Take default assumptions in science. At the theoretical level, the task is to identify which assumptions are likely to be correct given current evidence and theory (we can call these our *actual hypotheses*). At the practical level, the task is to identify which assumptions are likely to drive scientific progress in particular cases, in part by supporting established research programs and in part by opening new ones (we can call these our *working hypotheses*). Overall, the aim is to balance theoretical rigor with practical progress, recognizing that assumptions work differently when taken as truth claims and when taken as mere tools.

We can make a similar distinction in ethics. At the theoretical level, the task is to identify which assumptions ideally balance the risks associated with false positives (that is, what happens when we mistakenly treat objects like subjects) and the risks associated with false negatives (that is, what happens when we mistakenly treat subjects like objects) (we can call these our *ideal hypotheses*). At the practical level, the task is to identify which assumptions are likely to drive ethical progress at present, in part by building momentum toward a better calibrated moral circle in the future (we can call these our *non-ideal hypotheses*). Overall, here too the aim is to balance aspiration with tractability, recognizing that highly inclusive proposals can easily backfire, while highly exclusionary ones can easily perpetuate avoidable harms.

There are a lot of interesting connections between these four general purposes for making default assumptions. In both science and ethics, the best assumptions at the level of theory may tend to be more static, since they seek to capture what is true or right given current information and arguments. By contrast, the best assumptions at the level of practice may tend to be more dynamic and contextual, since they must adapt to the shifting capacities and priorities of imperfect agents and institutions. In both cases a division of labor between theory and practice can be helpful, since it allows our theoretical assumptions to provide stability and our practical assumptions to provide flexibility. But it can also lead to tensions within and across both science and ethics, and these tensions must be managed with care.

Another connection concerns how expansive our assumptions should be. The best assumptions at the level of scientific theory and ethical practice may tend to be more exclusionary, since they tend to be constrained by the demand for evidence and feasibility, respectively. By contrast, the best assumptions at the level of scientific practice and ethical theory may tend to be more inclusive, since they can look beyond what we take to be correct and realistic at present, respectively. Here, too, this division of labor can be helpful, since incrementalism in some areas can prevent backfire effects while radicalism in other areas can prevent lock-in effects. Yet tensions can emerge here as well, and striking a balance may require attending at least somewhat to practical concerns in research contexts and to theoretical concerns in policy contexts.

Still, despite these differences, a similarity across all four purposes is that a universal policy of denying nonhuman consciousness unless strong evidence supports it is often problematic. In science, such a policy can limit our ability to represent uncertainty about consciousness and make progress toward a secure theory. In ethics, it can limit our ability to mitigate welfare risks and make progress on moral circle expansion. While we should presumably avoid attributing full consciousness to all of the entities discussed in this paper in all contexts, we should at least attribute *some* consciousness to *many* of these entities in *many* contexts. Where possible, we should also use probability estimates to strike a balance, since probability estimates are useful for modeling our uncertainty and determining proportionality in policymaking.

The stakes of our default assumptions about the distribution of consciousness are high. While researchers are making progress toward a secure theory of consciousness, we are not yet on the cusp of achieving this goal. And as progress continues, our default assumptions about the distribution of consciousness could shape our decisions in a range of contexts, determining the trajectory of consciousness science and the fates of countless entities. In this paper I made a series of distinctions and observations that I hope can be useful in this predicament. But they will not be enough. Further research is needed before we can responsibly determine what to assume about different kinds of entities — from plants and fungi to robots and chatbots — for different purposes — from research and education to food and infrastructure policy.

## Footnotes

1 This is an invited article for a special issue.

2 Sober (2005) attributes the preference for false negatives in this context to a scientifically ingrained but dubious principle known as Morgan’s Canon — the idea that animal behavior should be explained by the simplest, or “lowest,” cognitive processes (Morgan, 1894). Additionally, LeDoux (2020) notes that many surprisingly sophisticated human behaviors can be accomplished without consciousness, suggesting that similar nonhuman behaviors can be performed without consciousness as well. This is not merely a methodological point but a substantive one; see note 11 for more on this distinction. However, Morgan’s canon — along with the idea that behaviors that *can* be explained without consciousness *should* be so explained — has lately received increasing criticism (Fitzpatrick, 2008, 2017; Mikhalevich et al., 2017; Starzak, 2017; Dacey, 2016; Meketa, 2014; Sober, 2005).

3 As Dacey defines it, evidentialism is the view that “in no case should we endorse an explanation of animal behavior in terms of cognitive process X on the basis of the available evidence if that evidence gives us no reason to prefer it to an alternative explanation in terms of a different cognitive process Y—whether this be lower or higher on the ‘psychical scale’” (Dacey, 2023).

4 For a summary of criticisms of null hypothesis testing, see, e.g. Morgan (2002), Schoot et al. (2011), Nickerson (2000), and Nix and Barnette (1998).

5 Bausman and Halina (2018) and Dacey (2023) argue that in fields like comparative psychology, default hypotheses are not technically “null.” That is, they are not statistical statements that can be falsified on the basis of data and mathematics. Rather, they are typically substantive explanations — usually the simplest explanations available. Thus, studies in comparative psychology do not technically use the null hypothesis testing methodology. I set that aside for now, but I do follow Dacey in using the term “default hypothesis” rather than “null hypothesis” for substantive explanations, while Bausman and Halina use the term “pseudo-null.”

6 See page 13 for a more detailed discussion of the precautionary principle and the expected weight principle.

7 The capacity for subjective experience is sometimes called “phenomenal consciousness” (Block, 1980). For more, see Nagel (1974) and Schwitzgebel (2016).

8 The idea that consciousness or sentience determines an individual’s moral status is called sentientism, and receives significant support. See, e.g., Birch (2024, chs. 2, 4), Nussbaum (2024), Roelofs (2023), Kagan (2022), Gibert and Martin (2022), Korsgaard (2018), Parfit (2011), DeGrazia (1996, 2021), Rawls (1971), and Bentham (1789).

9 Birch and others make a distinction between a *theory-heavy* approach, a *theory-neutral* approach, and a *theory-light* approach. A *theory-heavy* approach assumes particular theories of consciousness to explore their implications, whereas a *theory-neutral* approach assumes nothing at all. A *theory-light* approach strikes a balance by making minimal assumptions, consistent with multiple leading theories of consciousness, and exploring their implications. For more, see de Weerd (2024), Birch (2022), and Shevlin (2021).

10 For more on integrated information theory, see Chalmers (1996, pp. 276–308). For more on representational theories of consciousness, see Tye (2000, 1995).

11 There is a potential ambiguity in what “default assumption” means at the theoretical level. One interpretation is methodological, concerning which general rule we should adopt (for instance, whether to affirm consciousness, deny consciousness, or assign a balanced prior before collecting evidence). By contrast, another interpretation is substantive, concerning what credence we should assign to a particular case before gathering case-specific evidence, given current theory and evidence (for instance, about related cases). Both interpretations matter. On the methodological reading, uncertainty about the nature of consciousness — amplified by the hard problem of consciousness and the problem of other minds — might itself warrant a non-skeptical prior rather than a near-zero one. By contrast, the substantive reading, our background knowledge — about theories of consciousness, evidence from related cases, a historical pattern of under-attribution, and other matters — might likewise support non-skeptical priors in many cases. When I suggest that the “traditional skeptical assumption” may be too restrictive, then, I mean that it may be too restrictive on either or both interpretations: as a methodological rule under deep uncertainty about consciousness and as a substantive starting credence for new cases given current background knowledge. That said, my primary focus here is on both of these points combined: Given uncertainty about consciousness *and* current background knowledge, balanced starting points for many new cases are warranted.

12 In upcoming work, Derek Shiller and colleagues explore a range of prior probabilities. They find that, when combined with the evidence, priors above 10% seem to produce the most plausible posterior probabilities for familiar systems like humans and chickens (correspondence).

13 Put more generally, it might make sense to assign a higher probability to beings with physical features that resemble relevant physical features in humans. De Weerd (2024) makes a similar argument in favor of searching for theory-informed markers of consciousness.

14 Many researchers hold that the methodology of null hypothesis testing was itself created as a practical tool for advancing science, which makes the methodology both theoretical and practical (Mikhalevich, 2015; Godfrey-Smith, 1994). According to Godfrey-Smith (1994, 280-282), the original “accept/reject procedure for experimental hypotheses” was “intended as a behavioral strategy, where ‘accepting’ a hypothesis meant acting as if the hypothesis were true.”

15 A related way that an assumption can have practical value is by playing a role in an argument. For instance, Sebo and Long (2023) argue that even if we make conservative assumptions about the nature of consciousness and the future of AI, we should still take there to be a non-negligible chance of near-future AI consciousness. In this case, Sebo and Long are making these assumptions neither because they expect them to be correct nor because they expect them to spur innovation, but rather because they want to show that inclusive conclusions can flow even from exclusionary premises.

16 For more on theoretical vs. practical reason, see, e.g., Dancy (2018), Korsgaard (1996), and Harman (1988).

17 Nick Bostrom has suggested that advanced AI could bring about human extinction, either because humans lose control of the technology or because we retain control and use it for bad purposes (see Bostrom and Cirkovic, 2011). Many experts continue to voice similar concerns, including Tegmark (2018) and Russell (2022). Setting aside the matter of consciousness, granting political rights to AI systems would arguably increase the risk of human extinction.

18 Kagan (2022) calls the kind of views expressed in this paragraph “practical realism.”

19 Or at least, incremental changes are more readily adopted by societal institutions, whereas transformative changes risk provoking resistance.

## References

[ref1] Adamska M. (2016). Sponges as models to study emergence of complex animals. Curr. Opin. Genet. Dev. 39, 21–28. doi: 10.1016/j.gde.2016.05.026, 27318691

[ref2] Aguilar W. Santamaría-Bonfil G. Froese T. Gershenson C. (2014). The past, present, and future of artificial life. Front. Robot. AI 1:8. doi: 10.3389/frobt.2014.00008

[ref3] Anderson D. R. Burnham K. P. Thompson W. L. (2000). Null hypothesis testing: problems, prevalence, and an alternative. J. Wildl. Manag. 64:912. doi: 10.2307/3803199

[ref9001] Andrews K. Birch J. Sebo J. (2025). Evaluating animal consciousness. Science 387, 822–824. doi: 10.1126/science.adp4990 39977511

[ref4] Andrews K. (2024). “All animals are conscious”: shifting the null hypothesis in consciousness science. Mind Lang. 39, 415–433. doi: 10.1111/mila.12498

[ref5] Andrews K. Birch J. Sebo J. Sims T. (2024) Background to the New York declaration on animal consciousness. Available online at: nydeclaration.com (Accessed October 22, 2025).

[ref6] Andrews K. Huss B. (2014). Anthropomorphism, anthropectomy, and the null hypothesis. Biol. Philos. 29, 711–729. doi: 10.1007/s10539-014-9442-2

[ref7] Augustine (1990). The trinity, vol. 5: New City Press.

[ref8] Banerjee D. Das P. K. Mukherjee J. (2023). “Nervous system” in Textbook of veterinary physiology (Singapore: Springer Nature), 265–293.

[ref9] Bausman W. Halina M. (2018). Not null enough: Pseudo-null hypotheses in community ecology and comparative psychology. Biol. Philos. 33:30. doi: 10.1007/s10539-018-9640-4, 41209429

[ref10] Bayne T. Shea N. (2020). Consciousness, concepts, and natural kinds. Philos. Top. 48, 65–84.

[ref11] Bentham J. (1789). An introduction to the principles of morals and legislation, vol. 45. New York: Dover Publications.

[ref12] Berkeley G. (1881). A treatise concerning the principles of human knowledge: J. B. Lippincott & Company.

[ref13] Birch J. (2017). Animal sentience and the precautionary principle. Anim. Sentience 2:1. doi: 10.51291/2377-7478.1200

[ref14] Birch J. (2022). The search for invertebrate consciousness. Noûs 56, 133–153. doi: 10.1111/nous.12351, 35321054 PMC7612530

[ref15] Birch J. (2024). The edge of sentience: risk and precaution in humans, other animals, and AI. Oxford: Oxford University Press.

[ref16] Birch J. Burn C. Schnell A. Browning H. Crump A. (2021) Review of the evidence of sentience in cephalopod molluscs and decapod crustaceans. LSE Consulting. LSE Enterprise Ltd, The London School of Economics and Political Science. Available online at: https://www.wellbeingintlstudiesrepository.org/af_gen/2 (Accessed October 22, 2025).

[ref17] Block N. (1980). “Troubles with functionalism” in The language and thought series (Cambridge: Harvard University Press), 268–306.

[ref18] Bostrom N. Cirkovic M. M. (Eds.) (2011). Global catastrophic risks. Oxford: Oxford University Press.

[ref19] Braunsdorf C. Mailänder-Sánchez D. Schaller M. (2016). Fungal sensing of host environment. Cell. Microbiol. 18, 1188–1200. doi: 10.1111/cmi.12610, 27155351

[ref20] Cabanac M. Cabanac A. J. Parent A. (2009). The emergence of consciousness in phylogeny. Behav. Brain Res. 198, 267–272. doi: 10.1016/j.bbr.2008.11.028, 19095011

[ref21] Carruthers P. (2000). Phenomenal consciousness: A naturalistic theory. Cambrudge: Cambridge University Press.

[ref22] Carruthers P. (2004). “The problem of other minds” in The nature of the mind. 1st ed (London: Routledge), 6–35.

[ref23] Chalmers D. J. (1995). Facing up to the problem of consciousness. J. Conscious. Stud. 2, 200–219.

[ref24] Chalmers D. J. (1996). The conscious mind: In search of a fundamental theory: Oxford University Press.

[ref25] Dacey M. (2016). The varieties of parsimony in psychology. Mind Lang. 31, 414–437. doi: 10.1111/mila.12113

[ref26] Dacey M. (2023). Evidence in default: rejecting default models of animal minds. Br. J. Philos. Sci. 74, 291–312. doi: 10.1086/714799

[ref27] Dancy J. (2018). Practical shape: A theory of practical reasoning. Oxford: Oxford University Press.

[ref28] de Waal F. B. M. (1999). Anthropomorphism and anthropodenial: consistency in our thinking about humans and other animals. Philos. Topics 27, 255–280. doi: 10.5840/philtopics199927122

[ref29] de Weerd C. R. (2024). A credence-based theory-heavy approach to non-human consciousness. Synthese 203:171. doi: 10.1007/s11229-024-04539-6

[ref30] DeGrazia D. (1996). Taking animals seriously: Mental life and moral status. Cambridge: Cambridge University Press.

[ref31] DeGrazia D. (2021). An interest-based model of moral status. Oxford: Oxford University Press.

[ref32] Deplazes A. Huppenbauer M. (2009). Synthetic organisms and living machines: positioning the products of synthetic biology at the borderline between living and non-living matter. Syst. Synth. Biol. 3, 55–63. doi: 10.1007/s11693-009-9029-4, 19816800 PMC2759422

[ref33] Descartes R. (1641/2008). “Second meditation” in Meditations on first philosophy (Oxford, Michael Moriarty (translator): Oxford University Press).

[ref34] Fitzpatrick S. (2008). Doing away with Morgan’s canon. Mind Lang. 23, 224–246. doi: 10.1111/j.1468-0017.2007.00338.x

[ref35] Fitzpatrick S. (2017). “Against Morgan’s canon” in The Routledge handbook of philosophy of animal minds. (Eds.) Kristin, A., and Jacob, B. (Oxford: Routledge).

[ref36] Furnham A. Boo H. C. (2011). A literature review of the anchoring effect. J. Socio-Econ. 40, 35–42. doi: 10.1016/j.socec.2010.10.008

[ref37] Gallup G. G. (1970). Chimpanzees: self-recognition. Science 167, 86–87. doi: 10.1126/science.167.3914.86, 4982211

[ref38] Gibert M. Martin D. (2022). In search of the moral status of AI: why sentience is a strong argument. AI Soc. 37, 319–330. doi: 10.1007/s00146-021-01179-z

[ref39] Godfrey-Smith P. (1994). Of nulls and norms. PSA Proc. Bienn. Meet. Philos. Sci. Assoc. 1994, 280–290. doi: 10.1086/psaprocbienmeetp.1994.1.193033

[ref40] Godfrey-Smith P. (2020). Gradualism and the evolution of experience. Philos. Topics 48, 201–220. doi: 10.5840/philtopics202048110

[ref41] Goff P. (2017). “Panpsychism” in The Blackwell companion to consciousness (West Sussex: John Wiley & Sons, Ltd).

[ref42] Gómez-Márquez J. (2021). What is life? Mol. Biol. Rep. 48, 6223–6230. doi: 10.1007/s11033-021-06594-5, 34318436 PMC8376694

[ref43] Harman G. (1988). Change in view: Principles of reasoning. Cambridge: MIT Press.

[ref44] Kagan S. (2022). “How to count animals, more or less” in Uehiro series in practical ethics (Oxford University Press).

[ref45] Kaufmann A. (2024). All animals are conscious in their own way: comparing the markers hypothesis with the universal consciousness hypothesis. Front. Psychol. 15:1405394. doi: 10.3389/fpsyg.2024.1405394, 38803831 PMC11128545

[ref46] Knauff M. Wolf A. G. (2010). Complex cognition: the science of human reasoning, problem-solving, and decision-making. Cogn. Process. 11, 99–102. doi: 10.1007/s10339-010-0362-z, 20309605

[ref47] Knutsson S. Munthe C. (2017). A virtue of precaution regarding the moral status of animals with uncertain sentience. J. Agric. Environ. Ethics 30, 213–224. doi: 10.1007/s10806-017-9662-y

[ref48] Korsgaard C. M. (1996). The sources of normativity. Cambrdige: Cambridge University Press.

[ref49] Korsgaard C. (2018). Fellow creatures: Our obligations to the other animals. Oxford: Oxford University Press.

[ref50] Kruschke J. K. (2010). What to believe: Bayesian methods for data analysis. Trends Cogn. Sci. 14, 293–300. doi: 10.1016/j.tics.2010.05.001, 20542462

[ref51] Kushwaha S. (2022). “Animals: form and function” in Introduction to biology: Biology as a natural science: The study of life in all its forms (Arcler Education Inc).

[ref52] Larson B. T. (2023). Perspectives on principles of cellular behavior from the biophysics of protists. Integr. Comp. Biol. 63, 1405–1421. doi: 10.1093/icb/icad106, 37496203 PMC10755178

[ref53] LeDoux J. (2020). The deep history of ourselves: The four-billion-year story of how we got conscious brains: Penguin.

[ref54] Long R. Sebo J. Butlin P. Finlinson K. Fish K. Harding J. . (2024). Taking AI welfare seriously. arXiv preprint arXiv:2411.00986.

[ref55] Meketa I. (2014). A critique of the principle of cognitive simplicity in comparative cognition. Biol. Philos. 29, 731–745. doi: 10.1007/s10539-014-9429-z

[ref56] Mikhalevich I. (2015). Experiment and animal minds: why the choice of the null hypothesis matters. Philos. Sci. 82, 1059–1069. doi: 10.1086/683440

[ref57] Mikhalevich I. Powell R. Logan C. (2017). Is behavioural flexibility evidence of cognitive complexity? How evolution can inform comparative cognition. Interface Focus 7:20160121. doi: 10.1098/rsfs.2016.0121, 28479981 PMC5413892

[ref58] Monton B. (2019). How to avoid maximizing expected utility. Philos. Imprint 19, 1–25.

[ref59] Morgan C. L. (1894). An introduction to comparative psychology, vol. 27. London: W. Scott.

[ref60] Morgan G. A. (2002). Problems with null hypothesis significance testing (NHST): What do the textbooks say? PhilPapers. Available online at: https://philpapers.org/rec/MORPWN (Accessed October 22, 2025).

[ref61] Nagel T. (1974). What is it like to be a bat? Philos. Rev. 83:435. doi: 10.2307/2183914

[ref62] Nickerson R. S. (2000). Null hypothesis significance testing: a review of an old and continuing controversy. Psychol. Methods 5, 241–301. doi: 10.1037/1082-989X.5.2.241, 10937333

[ref63] Nix T. W. Barnette J. J. (1998). The data analysis dilemma: ban or abandon. A review of null hypothesis significance testing. *Research in the Schools*, 5, 3–14.

[ref64] Nussbaum M. C. (2024). Justice for animals. New York: Simon & Schuster.

[ref65] Panda T. Mishra N. Rahimuddin S. Pradhan B. Mohanty R. (2025). Beyond silence: a review—exploring sensory intelligence, perception and adaptive behaviour in plants. J. Bioresour. Manag. 12.

[ref66] Parfit D. (2011). On what matters, vol. 1: Oxford University Press.

[ref67] Prusiner S. B. (1984). Prions. Sci. Am. 251, 50–59. doi: 10.1038/scientificamerican1084-50, 6385236

[ref68] Rawls J. (1971). A theory of justice. Belknap Press.

[ref69] Roelofs L. (2023). Sentientism, motivation, and philosophical vulcans. Pac. Philos. Q. 104, 301–323. doi: 10.1111/papq.12420

[ref70] Russell S. (2022). Artificial intelligence and the problem of control. Perspect. Digit. Humanism 19, 1–322. doi: 10.1007/978-3-030-86144-5_3

[ref9003] Schoot R. Van de Hoijtink H. Romeijn J.-W. (2011). Moving beyond traditional null hypothesis testing: Evaluating expectations directly. Front. Psychol. 2. doi: 10.3389/fpsyg.2011.00024 PMC3111216 21713172

[ref71] Schwitzgebel E. (2016). Phenomenal consciousness, defined and defended as innocently as I can manage. J. Conscious. Stud. 23, 224–235.

[ref72] Sebo J. (2018). The moral problem of other minds. Harv. Rev. Philos. 25, 51–70. doi: 10.5840/harvardreview20185913

[ref73] Sebo J. (2023). Esoteric altruism: does effective altruism require its own destruction? Georget. J. Law Public Policy 21.

[ref74] Sebo J. (2025). The moral circle: Who matters, what matters, and why. New York: W.W. Norton.

[ref75] Sebo J. Long R. (2023). Moral consideration for AI systems by 2030. AI Ethics 5, 591–606. doi: 10.1007/s43681-023-00379-1

[ref76] Seth A. K. Baars B. J. Edelman D. B. (2005). Criteria for consciousness in humans and other mammals. Conscious. Cogn. 14, 119–139. doi: 10.1016/j.concog.2004.08.006, 15766894

[ref77] Shevlin H. (2021). Non-human consciousness and the specificity problem: a modest theoretical proposal. Mind Lang. 36, 297–314. doi: 10.1111/mila.12338

[ref78] Skinner B. F. (1953). Science and human behavior. New York: Simon & Schuster.

[ref79] Sober E. (2005). “Comparative psychology meets evolutionary biology: Morgan’s canon and cladistic parsimony” in Thinking with animals: New perspectives on anthropomorphism. eds. Mitman G. Datson L. (New York: Columbia University Press).

[ref80] Sober E. (2008). Evidence and evolution: The logic behind the science. Cambridge: Cambridge University Press.

[ref81] Starzak T. (2017). Interpretations without justification: a general argument against Morgan’s canon. Synthese 194, 1681–1701. doi: 10.1007/s11229-016-1013-4

[ref82] Tegmark M. (2018). Life 3.0: Being human in the age of artificial intelligence. New York: Vintage.

[ref84] Tye M. (1995). Ten problems of consciousness: A representational theory of the phenomenal mind. Cambridge: MIT press.

[ref85] Tye M. (2000). Consciousness, color, and content. Cambridge: MIT Press.

[ref86] Tye M. (2016). Tense bees and shell-shocked crabs: Are animals conscious? New York, US: Oxford University Press.

[ref87] Schoot R. Van de Hoijtink H. Romeijn J.-W. (2011). Moving beyond traditional null hypothesis testing: evaluating expectations directly. Front. Psychol. 2::24. doi: 10.3389/fpsyg.2011.00024 21713172 PMC3111216

[ref88] Varner G. E. (2012). Personhood, ethics, and animal cognition: Situating animals in hare’s two-level utilitarianism. New York: Oxford University Press.

[ref89] Veit W. (2023). A philosophy for the science of animal consciousness. New York: Routledge.

[ref90] Watson J. B. (1928). The ways of behaviorism. New York: Harper & Brothers.

